# The roles of CD24-Sec14 like lipid binding 2 (SEC14L2) axis in the neoplastic progression of oral squamous cell carcinomas

**DOI:** 10.1016/j.jds.2025.08.014

**Published:** 2025-08-22

**Authors:** Shi-Rou Chang, Chung-Hsien Chou, Chung-Ji Liu, Kuo-Wei Chang, Jian-Hua Pan, Sheng-Lin Yen, Shu-Chun Lin

**Affiliations:** a Institute of Oral Biology, College of Dentistry, National Yang Ming Chiao Tung University, Taipei, Taiwan; b Department of Dentistry, College of Dentistry, National Yang Ming Chiao Tung University, Taipei, Taiwan; c Department of Stomatology, Taipei Mackay Hospital, Taipei, Taiwan; d Department of Stomatology, Taipei Veterans General Hospital, Taipei, Taiwan

**Keywords:** CD24, Head and neck carcinoma, Oral carcinoma, SEC14L2

## Abstract

**Background/purpose:**

Immune stimulation or escape are critical factors determining the survival of malignancies, including oral squamous cell carcinoma (OSCC) and head and neck SCC (HNSCC). CD24 (CD24A in mice) is a glycoprotein anchored to cell membranes, modulating macrophages' immune responses, cell interaction, tumorigenesis, and stemness. However, its roles in the OSCC pathogenesis are still controversial. The modulation of CD24 on the oncogenicity and immunity of OSCC were investigated in this study.

**Materials and methods:**

Knockdown approaches and the establishment of stable tet-off CD24 expression cell subclones were used in cell and mouse models for phenotypic and transcriptomic analysis. Bioinformatic assessments were performed to specify the clinicopathological implications.

**Results:**

CD24 expression modulated the increase of migration and invasion and the upregulation of phosphatidylcholine/phosphatidylinositol transfer protein Sec14 like lipid binding 2 (SEC14L2) expression. The syngeneic grafts of CD24 tet-off expressing murine OSCC cell subclones exhibited modest changes of immune cell infiltration within tumors and were devoid of immune profile disruption in the recipient's neck lymph node and spleen. The shutdown of CD24 with doxycycline treatment drastically suppressed the growth of CD24 tet-off tumors. A correlation between CD24 expression and myeloid dendritic cell population was noted in murine and human OSCC tissue. Concordances in CD24 and SEC14L2 expression and oncogenic induction were noted in murine tumors. SEC14L2 was upregulated in HNSCC/OSCC tumors, and it was an unfavorable survival predictor.

**Conclusion:**

This study's elucidation of the oncogenic potential of the CD24-SEC14L2 axis may signify the therapeutic efficacy of CD24 targeting for HNSCC/OSCC.

## Introduction

Oral squamous cell carcinoma (OSCC) is one of the highly prevalent malignancies in the world, accounting for a significant proportion of head and neck cancers (HNSCC).^1^ OSCC primarily affects the epithelial cells lining the oral cavity, including the lips, tongue, floor of the mouth, and buccal mucosa. Despite advances in treatment, OSCC remains a major global health concern due to its high mortality rate and tendency for late-stage diagnosis.^1^ Risk factors for OSCC include areca chewing, tobacco use, alcohol consumption, and infection with high-risk strains of human papillomavirus (HPV).^2^^,^^3^ Aberrant expression of oncogene molecules such as miR-31, miR-146a, and NEAT1 promote HNSCC/OSCC progression by targeting downstream factors.^4^, ^5^, ^6^, ^7^, ^8^ miR-146a is also a master immune/inflammation regulator by activating NFκB pathways.^9^ Although risk factors and genetic abnormalities contribute to its development, immune cell signatures also define the progression of tumors and disease outcomes.^10^^,^^11^

CD24 (also named CD24A in the mouse genome) is a highly glycosylated, GPI-anchored cell surface protein predominantly expressed on immune cells and specific epithelial and neuronal cells.^12^ Studies have demonstrated that the overexpression of CD24 in cancer cell lines enhances cell proliferation and adhesion through integrin activation.^13^ CD24 expression is upregulated in many cancers, and research suggests that CD24 functions as an oncogene, promoting cell growth, migration, and invasion.^14^^,^^15^ Abrogation of CD24 expression in human cancer cells has been associated with decreased cell growth and migration capabilities.^16^, ^17^, ^18^, ^19^, ^20^ CD24 is also crucial in regulating tumor immune responses.^14^^,^^15^ In the tumor microenvironment, CD24 on the surface of tumor cells binds to Siglec-10 on macrophages, which misguides macrophage recognition and prevents the initiation of phagocytosis.^14^, ^15^, ^16^^,^^21^ CD24 is often overexpressed in various human cancers, including HNSCC/OSCC,^15^^,^^22^, ^23^, ^24^, ^25^ and its expression is positively correlated with poor prognosis.^22^^,^^25^ Animal studies have confirmed that anti-CD24 therapy reduces the growth of SCC grafts and stimulates T-cell responses in tumors.^25^ However, a study has also shown the downregulation of CD24 expression in OSCC. CD24 is a favorable prognostic predictor of OSCC, and it hampers myeloid-derived suppressor cell (MDSC) activity.^26^ A study deciphered that higher CD24 levels in OSCC cells inactivate AKT and attenuate β-catenin.^27^ The controversial roles of CD24 in OSCC progression and immune modulation require elucidation.

The SEC14L2 (Sec14 like lipid binding 2) gene encodes a phosphatidylcholine/phosphatidylinositol transfer protein involved in lipid transport . SEC14L2 plays an important role in various cellular functions, especially in the liver, where its expression is related to fat metabolism and cholesterol synthesis.^28^ SEC14L2 can regulate lipid metabolic pathways and inhibit the growth and proliferation of cancer cells.^29^ In hepatocellular carcinoma (HCC), expression of SEC14L2 is associated with the effect of antiviral drugs on hepatocytes and, in some cases, can enhance the therapeutic effect of the drugs.^30^ The SEC14L2 expression was significantly higher in OSCC and HNSCC than in adjacent normal tissues.^31^ Upregulation of SEC14L2 was associated with advanced tumor stage, grade, and metastasis in cancer patients. Besides, the neo-antigenic SEC14L2 peptide identified from the breast cancer mediated the anti-tumor T cell functions.^32^ The functional roles of SEC14L2 in HNCC/OSCC remain obscure.

This study utilized CD24 knockdown and tet-off-CD24 overexpression systems in murine OSCC cell lines to further explore the oncogenic role of CD24 in oral carcinogenesis and its contribution to immune dysregulation.^33^ Additionally, we investigated the regulation of downstream effector SEC14L2 and its involvement in CD24-mediated oncogenesis.

## Materials and methods

### Cells

The murine OSCC cell lines MOC-L1 and MTCQ1 we established were cultured as previously described.^34^, ^35^, ^36^ MOC-L1 and MTCQ1 were tumorigenic lines, and MTCQ1-GFP is a subclone of MTCQ1 tagged with GFP. The cells were cultivated in complete DMEM medium (Thermo Scientific, Waltham, MA, USA) containing 10 % fetal bovine serum (Biological Industries, Kibbutz Beit Haemek, Israel), 1 % pen-strep-amp antibiotics (Biological Industries), and 2 mM l-glutamine (Biological Industries).

### Phenotypic assays

Cells were seeded into 96-well plates at a density of 2500 cells per well, and the cell proliferation over the following 4 days was analyzed by MTT assay. The cell migration and invasion assays were performed using 8 μm 24-well Transwell chambers (Merck Millipore, Billerica, MA, USA). For the migration assays, cells were collected and seeded into the upper chamber of a Transwell at a density of 1 × 10^5^ cells per well. For the invasion assays, 50 μl of 10 % Matrigel (BD Biosciences, San Jose, CA, USA) was used to coat each Transwell chamber, and then 2 × 10^5^ cells were seeded onto the Matrigel-coated Transwell. After incubating at 37 °C for 48 h, the Transwell membranes were fixed and stained with 10 μg/mL Hoechst 33258.^34^, ^35^, ^36^ Images of the migrated or invading cells were captured using a fluorescence microscope. The cell numbers in each picture were counted and then normalized to allow the fold change to be calculated.

### siRNA

Pilot tests optimized that the treatment with 60 nM si-CD24 or si-SEC14L2 for 48 h rendered effective induction or knockdown of expression. The reagents are described in Table S1. TransFectin lipid reagent (BioRad, Hercules, CA, USA) was used for transfection. Unless otherwise specified, all other reagents were purchased from Sigma–Aldrich (St Louise, MO, USA).

### RNA extraction and qPCR analysis

Cells were collected in TriPure Isolation Reagent (Roche, Basel, Switzerland) and disrupted using 1-bromo-3-chloropropane. The lysate was collected, and the upper aqueous phase containing the RNA was transferred to be mixed thoroughly with isopropanol and then spun at maximum speed to pellet the precipitated RNA. The resulting pellet was washed and then dissolved in nuclease-free water. The threshold cycle (C_t_) method was used for qPCR analysis.^5^ The primers or probes for qPCR analysis are listed in Table S2. The resulting information was analyzed using the –ΔΔC_t_ method, and values were calculated relative to internal controls.

### Western blot analysis

Western blot analysis followed the approach of previous protocols.^5^ Signals were revealed by Western Lightning Chemiluminescence Reagent Plus kit (Thermo Scientific, Waltham, MA, USA) and detected using a FUJIFILM LAS-4000 mini luminescent image analyzer (GE Life Sciences, Piscataway, NJ, USA). The signals of CD24 were normalized against GAPDH to designate the expression level.

### Plasmid construction and stable cell subclone establishment

A 55-bp multiple cloning site (MCS) fragment was engineered into a tet-off lentiviral vector (pCW57.1, plasmid #41393, Addgene, Watertown, MA, USA) to generate a 7470-bp vector designated tet-off-MCS. An amplicon encompassing the 219-bp cDNA of murine CD24 coding sequence was digested with Bam HI and EcoR I (Table S2) and ligated into the tet-off-MCS. OSCC cell clones that stably express CD24 were established after viral infection and blasticidin selection and named TetOFF-CD24. The control cell clones were TetOFF-VA.

### Tumor cell grafts

We inspected the infiltration of immune cells in primary tumors, the host's loco-regional lymph node, and the spleen. 6 × 10^6^ TetOFF-CD24 and TetOFF-VA cells were injected into the buccal space of C57BL/6 mice (National Laboratory Animal Center, Taipei, Taiwan). Cells were 1:1 mixed with Matrigel (BD Biosciences) to give a total volume of 50 μl. Mice were sacrificed when their body weight loss was >1/3 or when the estimated tumor size exceeded 0.8 cm. Autopsies were performed to sample the tumor, the neck lymph node, and the spleen. The volume of the primary tumor and spleen were measured. RNAseq was performed on the primary tumor samples to achieve the transcriptome for immune state profiling and CD24 effector identification. Flow cytometry analysis was performed on node and spleen samples to investigate the changes in the immune cell population.

To investigate the growth of tumors as affected by CD24, 10^7^ TetOFF-CD24 cells were injected into the buccal space of C57BL/6 mice. When the tumor masses became discernible on the fifth day, the mice received daily doses of intraperitoneal Doxycycline (Doxy, 100 μg/g) for six consecutive days. The size of the tumor was recorded periodically. Five days after the termination of Doxy injection, mice were sacrificed, and tumors and adjacent tissues were harvested, imaged, dissected, and processed for RNA and protein analyses. The Institutional Animal Care and Use Committee (IACUC) of National Yang Ming Chiao Tung University approved the animal study.

### Flow cytometry

The node and spleen tissues were cut into pieces and placed in the tube for dissociation. Facilities and consumables, including Tumor Dissociation Kit (MACS®, 130-096-730), gentleMACS C Tubes (130-093-237), and gentleMACS™ Dissociator (130-093-235) supplied by Miltenyi Biotec (San Jose, CA, USA) were used to convert node or spleen tissues into single cells. After the dissociator was exposed to 37 °C for 45 min, the liquid was extracted and transferred to MACS SmartStrainers (70 μm) (130-098-462) to achieve single cells. Aliquots of the cell suspension were evaluated to ensure cell presence. Dissociated or cultivated cells were resuspended in 2 mL of 0.1 % BSA for blocking, washing, and stained with appropriate antibodies according to the manufacturer's recommendations (Table S3). The samples were then processed on the flow cytometer (Beckman Coulter Cytoflex, Brea, CA, USA) to sort the immune cell population according to the flow chart listed in Table S4. The immune cell populations, specifically CD8+ cytotoxic T cells, CD4+ helper T cells, Treg, M1 macrophages, M2 macrophages, and MDSC, were assessed using flow cytometry.

### HNSCC/OSCC transcriptional signatures

The patient information for HNSCC and normal tissue information was achieved from The Cancer Genome Atlas (TCGA, https://portal.gdc.cancer.gov/). 100 OSCC tumors (from 99 patients) and 33 noncancerous matched normal tissue samples were collected at Taipei Mackay Memorial Hospital (Table S5). This study was approved by the ethics reviewing committee with an approval number of 18MMHIS187e. Written informed consent was obtained from patients prior to sampling. Qualified RNAs were subjected to RNA-Seq. Transcripts per kilobase of transcript per million (TPM) mapped reads designated the clean reads of RNA-Seq.

### RNA-Seq and *in silico* analysis

After homogenizing the murine tumor tissues under frozen status, the qualified RNA was subjected to RNA-Seq using the platforms established in the National Genomics Center for Clinical and Biotechnological Applications in National Yang Ming Chiao Tung University. The DESeq program was used to retrieve differentially expressed transcripts. CIBERSOR-ABS, EPIC, MCP counter, QUANTISEQ, TIMER, and XCELL algorithms annotated the immune microenvironment.

### Statistical analyses

The data were presented as means ± SE. Mann–Whitney tests, unpaired t-tests, two-way ANOVA tests, correlation analysis, Cox regression analysis, and Kaplan–Meier survival analysis were used to compare the differences between the various groups of results. A *P*-value of less than 0.05 was considered statistically significant.

## Results

### Knockdown of CD24 decreases the migration and invasion of cells

**Figure 1:**
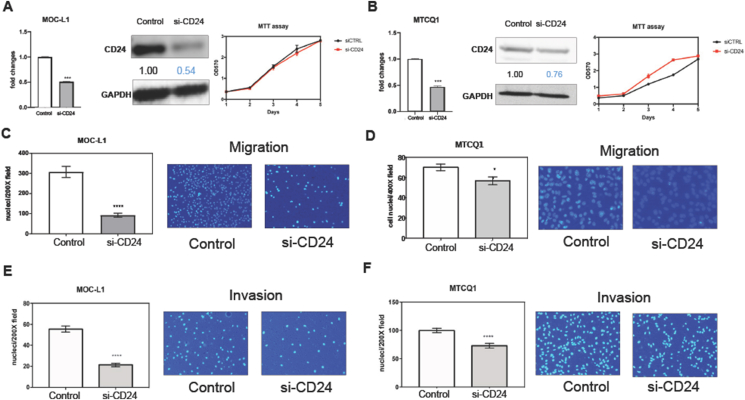
Knockdown of CD24 expression. Cells are treated with 60 nM si-CD24 oligonucleotide or control oligonucleotide for 48 h (A, C, E) MOC-L1 cell line. Triplicate analysis. (B, D, F) MTCQ1 cell line. Triplicate analysis. (A, B) Lt, CD24 mRNA expression; middle, CD24 protein expression; Rt, growth curve revealed by MTT assay (Y axis, ratio of OD_570_ reading). (C, D) migration assay. Lt, quantification of migrated cell; Rt, a representative field of migrated cell on transwell membrane. x200x or x400. (E, F) invasion assay. Lt, quantification of invaded cell; Rt, a representative field of invaded cell on transwell membrane. x200. Values below the Western blot diagram denote normalized expression levels. ∗, ∗∗∗, and ∗∗∗∗, *P* < 0.05, *P* < 0.001, and *P* < 0.0001, respectively.

MOC-L1 and MTCQ1 cell lines were treated with si-CD24 and a scrambled control. Analysis of RNA and protein harvested after transfection verified the knockdown of CD24 (Fig. 1A and B, Lt and middle). Knockdown of CD24 did not influence cell growth (Fig. 1A and B, Rt). However, it significantly decreased cell migration (Fig. 1C and D) and invasion (Fig. 1E and F).

### Exogenous CD24 expression increases the migration and invasion of cells

**Figure 2:**
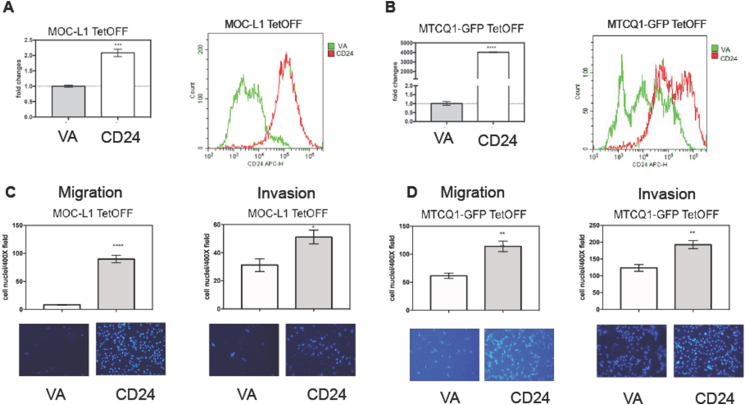
Exogenous CD24 expression. MOC-L1 TetOFF and MTCQ1-GFP TetOFF cell subclones are analyzed. (A, C) MOC-L1 TetOFF-CD24 and MOC-L1 TetOFF-VA. Triplicate analysis. (B, D) MTCQ1-GFP TetOFF-CD24 and MTCQ1-GFP TetOFF VA. Triplicate analysis. (A, B) Lt, CD24 mRNA expression. Rt, fluorescent CD24 level revealed by flow cytometry. (C, D) Upper Lt and Upper Rt, quantification of migrated cells and invaded cells, respectively. Lower Lt and Lower Rt are representative fields of migrated and invaded cells. ×200 or x400. ∗, ∗∗, ∗∗∗, and ∗∗∗∗, *P* < 0.05, *P* < 0.01, *P* < 0.001, and *P* < 0.0001, respectively.

To evaluate the effect of CD24 overexpression on migration and invasion, stable cell clones of MOC-L1 and MTCQ1-GFP harboring the TetOFF vector system were established by lentiviral infection and selection. The CD24 overexpression in the cell lines was confirmed by qPCR and flow cytometry analysis (Fig. 2A and B). In both cell lines, a significant increase in migration and invasion abilities was observed (Fig. 2C and D). These findings signify the pronounced effects of CD24 on the migratory phenotype.

### Tumors harboring CD24 expression exhibit disrupted immune cell infiltration

**Figure 3:**
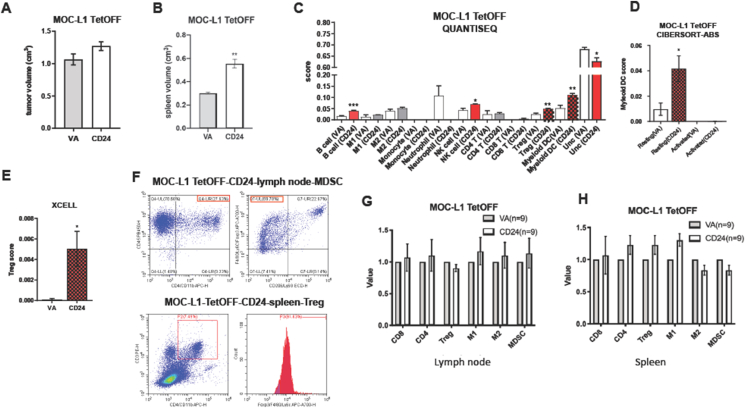
Analysis of MOC-L1 TetOFF-CD24 and MOC-L1 TetOFF-VA tumors. (A). Tumor volume. (B) Spleen volume. (C–E) Algorithmic analysis. QUNTISEL, a representative part of CIBERTSORT-ABS, and a representative part of XCELL, respectively. (A–E) Five samples are in each group. (F) Representative flow cytometric analysis of MDSC in a lymph node (Upper) and Treg in a spleen from a MOC-L1 TetOFF-CD24 mouse. (G, H) Lt, lymph node. Rt, spleen. Quantification of CD8, CD4, Treg, M1 macrophage, M2 macrophage, and MDSC in MOC-L1 TetOFF tumors. Nine samples are in each group. The values of CD24 samples are normalized with the average value of VA samples. ∗, and ∗∗, *P* < 0.05 and *P* < 0.01, respectively.

TetOFF-CD24 and TetOFF-VA MOC-L1 cell clones were orthotopically injected into the buccal space of C57BL/6 mice. Mice were sacrificed upon the growth of tumors to an estimated size exceeding 0.8 cm, as illustrated by the fluorescence image, or they lost one-third of their body weight. The tumor tissue, lymph node, and spleen were harvested for immune cell analysis. Although the tumor burden of VA and CD24 clones of MOC-L1 were not different (Fig. 3A), CD24 clones had a higher volume of spleen relative to VA controls (Fig. 3B). Due to the difficulties in achieving sufficient immune cell fraction from dissociated tumors for sorting, RNA-Seq was used to insight the transcriptome of tumors and the immune cell population. The immune cell population scores were analyzed using various algorithms. QUANTISEQ modules showed the increased B cell, NK cell, regulatory T cell (Treg), and Myleoid dendritic cell (DC) population and decreased uncharacterized cells in CD24 clones compared to controls (Fig. 3C). However, only the enrichment of resting Myleoid DC and Treg were also identified by other analytical modes (Fig. 3D and E). The immune cell populations, including CD8+ T cells, CD4+ helper T cells, Treg, M1 macrophages, M2 macrophages, and MDSC in the lymph node and spleen, were assessed using flow cytometry approaches. The representative sorting of Treg and MDSC was also illustrated (Fig. 3F). After normalizing with the mean percentage of cell population in controls, no significant change of immune cell population in the neck lymph node and spleen was noted in the mice harboring MOC-L1 tumors overexpressing CD24 (Fig. 3G and H).

**Figure 4:**
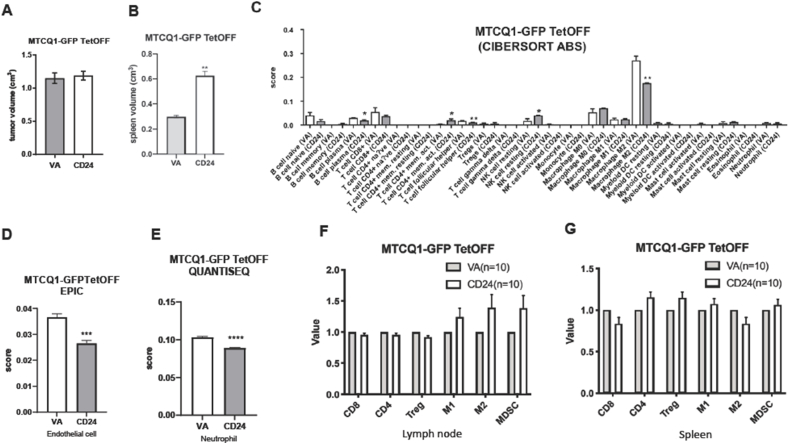
Analysis of MTCQ1-GFP TetOFF-CD24 and MTCQ1-GFP TetOFF-VA tumors. (A). Tumor volume. (B) Spleen volume. (C–E) Algorithmic analysis. CIBERTSORT-ABS, a representative part of EPIC, and a representative part of QUANTISEQ, respectively. (A–E) Four samples were in each group. (F, G) lymph node, and spleen, respectively. Quantification of CD8, CD4, Treg, M1 macrophage, M2 macrophage, and MDSC in MOC-L1 TetOFF tumors. Ten samples in each group. The values of CD24 samples are normalized with the average value of VA samples. ∗, ∗∗, ∗∗∗, and ∗∗∗∗, *P* < 0.05, *P* < 0.01, *P* < 0.001, and *P* < 0.0001, respectively.

In mice carrying the CD24 overexpression MTCQ1-GFP grafts, the spleen also enlarged despite the tumor burdens being equivalent to those of the controls (Fig. 4A and B). The decreased plasma cells, follicular T helper cells, M2 macrophages, endothelial cells, and neutrophils, and the increased memory T cells and NK cells resting were retrieved by only a solitary module (Fig. 4C–E). The immune cell population in the neck node and spleen of mice grafted with MTCQ1-GFP tumors overexpressing CD24 is not significantly different from controls (Fig. 4F and G).

### The induced shutdown of CD24 expression decreases the tumor growth

**Figure 5:**
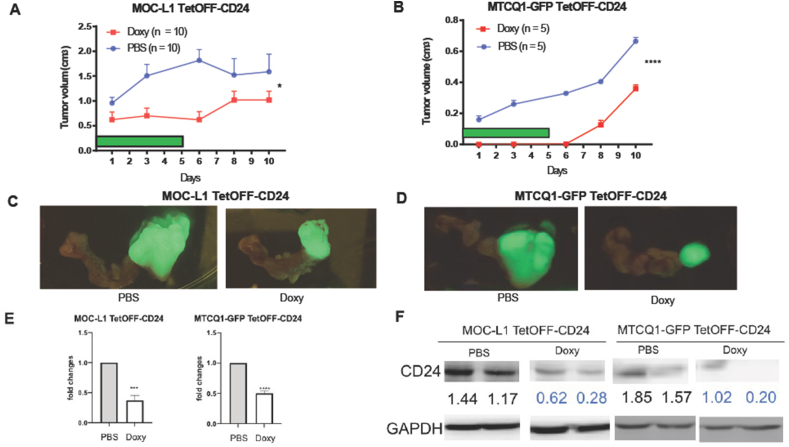
Tumorigenesis of TetOFF-CD24 cells and the induced shutdown of CD24 mediated by doxycycline. (A, C) MOC-LI TetOFF-CD24 tumors. Ten mice are in each group. (B, D). MTCQ1-GFP TetOFF-CD24 tumors. Five mice are in each group. (A, B) Growth curve. The green bar indicates the duration of the Doxy or PBS injection. (C, D) The fluorescence image of the harvested tumor and the connecting neck tissue. (E, F) Lt, MOC-LI TetOFF-CD24 tumors, Rt, MTCQ1-GFP TetOFF-CD24 tumors. (E) CD24 mRNA expression in tumors revealed by qPCR. Two or three samples in each group. (F) CD24 protein in tumors revealed by Western blot analysis. Values below the diagram indicate the normalized signals. Two samples in each group. ∗, ∗∗∗, and ∗∗∗∗, *P* < 0.05, *P* < 0.001, and *P* < 0.0001, respectively. Doxy, doxycycline.

Doxy treatment shuts down the expression of genes in the TetOFF vector. We optimized the dose and duration of Doxy treatment in pilot tests. When the TetOFF-CD24 grafts became discernible under fluorescence detection, Doxy was administered to mice six times to evaluate the potential of CD24 in affecting tumor growth. Control mice received PBS injections. The consecutive measurement of tumor growth s indicated that tumors in mice receiving Doxy injections were significantly smaller than controls (Fig. 5A and B). Tumors and connecting neck tissues were resected at the endpoint. Fluorescent imaging revealed the tumor lesion in the specimen (Fig. 5C). Analysis of representative tissues demonstrated the downregulation of CD24 mRNA expression and protein expression in tumors after the Doxy induction (Fig. 5D and E).

### SEC14L2 acts as a downstream effector of CD24

**Figure 6:**
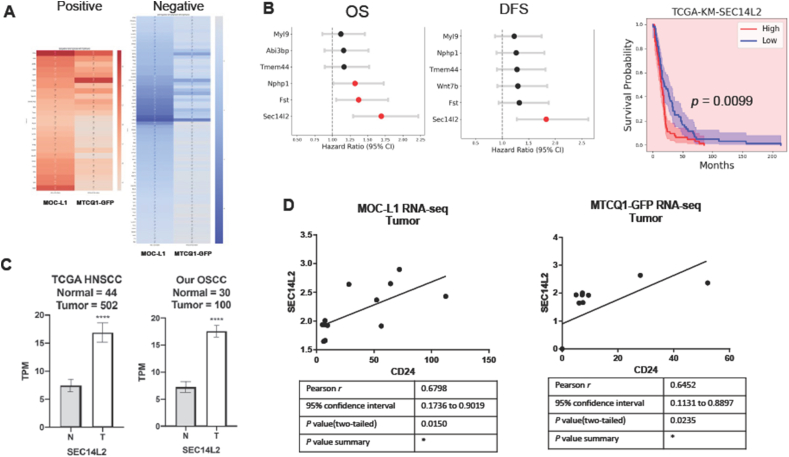
Identification of SEC14L2. RNA-Seq data of TetOFF-CD24 and TetOFF-VA tumors are processed to retrieve each cell line's transcriptomic profile. Four or five samples are in each group. (A). Heatmap of positively correlated transcripts (Lt) and negatively correlated transcripts as related to CD24 expression. (Rt). The gradient bar designates the *r* value. Red, positive; blue, negative. The detailed data are integrated in Table S6. (B) Survival analysis using the TCGA HNSCC dataset. Lt, Cox representative regression analysis of overall survival and disease-free survival. Rt, Kaplan–Meier plots for overall survival according to SEC14L2. These assays identify SEC14L2 as a survival predictor of HNSCC. (C) The expression of SEC14L2 in normal tissue and tumor. Lt, TCGA HNSCC dataset. Rt, our OSCC cohort. (D) Correlation of the TPM in MOC-L1 tumors (*n* = 10) and MTCQ1 tumors (*n* = 9). ∗∗∗, *P* < 0.0001. OS, overall survival; DFS, disease-free survival; N, normal; T, tumor.

DEseq analysis of the transcriptomic data was performed to retrieve 781 disrupted transcripts. Further dissection identifies 26 co-upregulated transcripts and 69 co-downregulated in MOC-L1 and MTCQ1-GFP tumors expressing CD24 in relation to controls (Fig. 6A, Table S6). Cox regression analysis revealed that SEC14L2, Fst, and Nphp1 expression were associated with unfavorable overall survival. Only SEC14L2 expression was associated with unfavorable disease-free survival in the TCGA HNSCC dataset (Fig. 6B, Lt and middle). Kaplan–Meier analysis confirmed SEC14L2 and Fst as poor survival predictors (Fig. 6B,Rt). SEC14L2 was highly upregulated in TCGA HNSCC tumor cohorts and our OSCC tumor cohorts compared to normal controls (Fig. 6C). SEC14L2 expression was correlated with CD24 expression in murine OSCC grafts (Fig. 6D and E).

**Figure 7:**
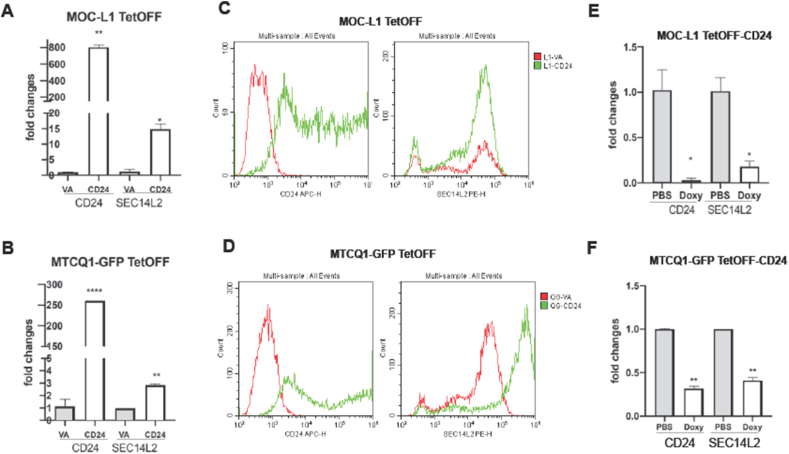
CD24 and SEC14L2 expression in murine cells and tumors. (A–C) MOC-L1 TetOFF. (B–D) MTCQ1-GFP TetOFF. (A–D) Cell lines. Triplicate analysis. (E, F) Tumors. Duplicate or triplicate analysis. (A, B, E, F) CD24 and SEC14L2 mRNA expression revealed by qPCR analysis. (C, D) CD24 and SEC14L2 protein levels are revealed by flow cytometry. Lt, CD24; Rt. SEC14L2, Upper, MOC-L1-TetOFF, Lower, MTCQ1-GFP TetOFF. It shows that the SEC14L2 expression levels follow the fluctuation of CD24 expression in cells and tumors. The level of CD24 repression in these two MOC-L1-TetOFF-CD24 tumors (in E) is more profound than that of the tumors shown in Fig. 5E. ∗, ∗∗, and ∗∗∗∗, *P* < 0.05, *P* < 0.01, and *P* < 0.0001, respectively.

MOCL-1 and MTCQ1 cells overexpressing CD24 showed a concordant upregulation of SEC14L2 mRNA expression (Fig. 7A and B). In this later passage batch of MOC-L1 cells, we also identify that the CD24 mRNA expression becomes much higher than in the early passage batch (Fig. 2A), suggesting a potential clonal expansion advantage associated with CD24. Flow cytometry analysis also showed the increased SEC14L2 cell fraction in MOCL-1 and MTCQ1-GFP cells overexpressing CD24 (Fig. 7C and D). The decreased CD24 mRNA expression induced by Doxy treatment in tumor grafts was also associated with the decreased SEC14L2 expression (Fig. 7E and F).

### Knockdown of SEC14L2 rescues the oncogenicity induced by CD24

**Figure 8:**
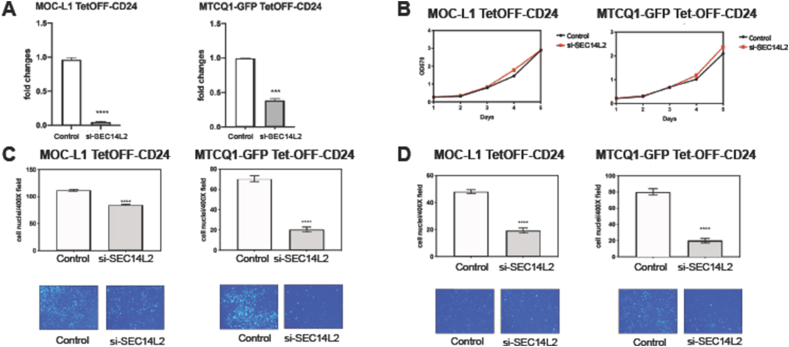
Knockdown of SEC14L2 rescues the CD24-mediated phenotypes. (A–D) MOC-L1 TetOFF-CD24 cell (Lt) and MTCQ1 TetOFF-CD24 cell (Rt) are treated with 60 nM si-SEC14L2 oligonucleotide for 48 h. Triplicate analysis. (A) SEC14L2 mRNA expression revealed by qPCR analysis. (B) The growth curve is revealed by the MTT assay (Y axis, ratio of OD_570_ reading). (C, D) migration assay and invasion assay, respectively. Upper quantification of migrated or invaded cells; Lower, a representative field of migrated or invaded cells on the transwell membrane. x200x or x400. ∗∗∗∗, *P* < 0.0001.

In the OSCC cells overexpressing CD24, the administration of si-SEC14L2 knocked down SEC14L2 mRNA expression (Fig. 8A). SEC14L2 knockdown did not change the growth of cells (Fig. 8B), while it decreased the migration and invasion of cells (Fig. 8C and D). The findings substantiated the presence of the CD24-SEC14L2 oncogenic axis in OSCC.

### The relevance of CD24 and SEC14L2 expression with the immune cell population in tumors

**Figure 9:**
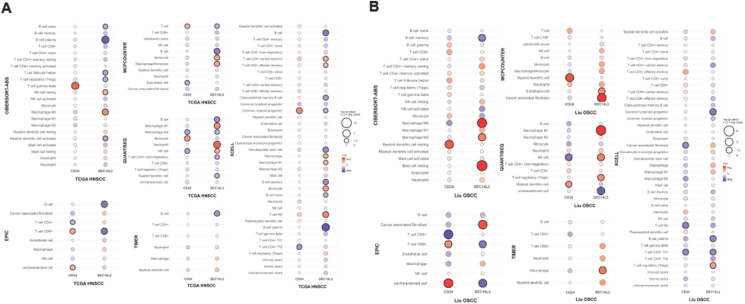
Immune cell signature associated with CD24 and SEC14L2 expression in human HNSCC/OSCC. According to the transcriptomic data, six different algorithms are used to elucidate the immune cell population, and the results are plotted as bubble plots. (A) TCGA HNSCC. (B) Our OSCC cohort. Bubbles designate the *r* values. Red, positively correlated. Blue, negatively correlated. CD24 and SEC14L2 bubbles are aligned in the Lt and Rt columns. A bubble with a thick border denotes a statistically significant correlation. Note the conspicuously positive correlation between CD24 and Myeloid DC and the negative correlation between SEC14L2 and B cell series in tumors.

The immune cell populations correlated with CD24 and SEC14L2 expression in TCGA HNSCC tumors, and our OSCC tumors were analyzed using multiple algorithms and summarized as bubble plots. The analysis of TCGA HNSCCs showed that CD24 had a positive correlation with CD8+T, Tγδ, monocyte, and myeloid progenitor and a negative correlation with NK, CD4+ helper T, endothelial cell, and M2 macrophage. SEC14L2 had a positive correlation with macrophage, monocyte, neutrophil, and T cell NK and an exclusively negative correlation with B cells (Fig. 9A). The analysis of OSCC tumors showed that CD24 had a positive correlation with Myeloid DC cell and uncharacterized cell, and a negative correlation with NK, T cell NK, CD4+ helper T cell, and CAF cell. SEC14L2 had a positive correlation with macrophage, Mast cell, and CAF and a negative correlation with B cell, CD8+T, lymphocyte progenitor, and uncharacterized cells (Fig. 9B). Pronounced Myeloid DC population associating with CD24 expression and decreased B cell and enriched CAF associating with SEC14L2 expression are present in HNSCC/OSCC tumors.

## Discussion

The combination of immune therapy with the conventional regimens is a current strategy for the interception of advanced OSCC.^37^ CD24 plays pluripotent roles in tumorigenesis, stemness, and tumor immune escape.^16^^,^^25^, ^26^, ^27^ Studies showed that CD24 mRNA expression is a favorable prognostic predictor of OSCC, and CD24 null mice are more susceptible to oral carcinogenesis.^26^ However, contradictory findings showed the upregulation of CD24 protein expression in OSCC, which defined a worse disease prognosis.^25^ Besides, targeting of CD24 attenuated the growth of SCC transplants and stimulated immune responses.^25^ To clarify the controversies, we have implemented knockdown and expression strategies on murine models to elucidate the modulation efficacies of CD24 in oncogenesis and tumor immunity. The association between CD24 and tumor aggressiveness is ascertained by *in vitro* functional approaches. The association is found between the inducible CD24 shutdown and the slower syngeneic orthotopic tumor growth, which further substantiates the linkage of CD24 to tumor progression. The findings substantiate the oncogenic roles of CD24 in OSCC, which are consistent with its upregulation in tumors and the poor prognosis of patients.^22^, ^23^, ^24^, ^25^ Although the discrepancy in the final tumor burden of control tumors and Doxy-induced tumors in MOC-L1 is not so eminent, which might be due to the growth of control tumors in the plateau phase or the shortage of Doxy induction, targeting of CD24 using inhibitors or monoclonal antibodies could be a promising strategy for OSCC interception.^25^

This study shows the diverse immune cell infiltration related to CD24 expression across MOCL-1 and MTCQ1 tumors, most likely due to the variation in CD24 expression level or tumor microenvironment in grafts. A study shows that MDSC activity and expansion being impeded by CD24 expression underlie the slow development of 4NQO-induced or orthotopically transplanted murine tongue SCC.^26^ However, such an effect could be the pan-CD24 effect since tumoral and immune cells have the same genetic background. Therefore, the solitary effects of tumor CD24 are undefined. The blockage of CD24 by intratumor injection of CD24mab attenuates the growth of subcutaneous SCC7 graft, a cell line established from the abdominal wall SCC of C3H mouse. Increased CD4+ and CD8+ and decreased tumor-associated macrophage infiltration in post-treatment tumors are noted.^25^ The consistent findings of Myeloid DC and Treg immune evasion factors being enriched in MOC-L1 tumors with CD24 overexpression may underscore the neoplastic progression.^38^ We have established a transcriptomic dataset encompassing 28 normal and 54 OSCC samples previously.^8^^,^^10^ This dataset has been expanded to include 33 normal and 100 OSCC samples in this study. The Myeloid DC is the only cell population repeatedly shown by multiple algorithms to positively correlate with CD24 expression in both the human TCGA HNSCC and the OSCC cohort. With the equivalent burden of primary tumors, the crucial immune components in the neck lymph node or spleen are not changed by the CD24 level. The findings raise concerns that the level of CD24 in tumors is not sufficient for evoking regional or systemic immune reactions. However, since the spleens of mice are enlarged following the CD24 expression, the disruption in immune or mesenchymal components in the node and spleen requires precise definition.

SEC14L2 is a lipid transfer protein, and an enzyme for lipid and cholesterol metabolism, which has been recently found to be involved in neoplastic pathogenesis and drug resistance.^28^, ^29^, ^30^, ^31^ This study provides fundamental cellular, preclinical, and clinical clues demonstrating SEC14L2 as an oncogenic downstream effector of CD24 in OSCC. The increase of CAF-like components in OSCC tumors having high SEC14L2 expression is substantiated by the knockdown experiments showing the modulation of SEC14L2 on migratory phenotypes. The consistent decrease of the B cell population found in HNSCC tumors exhibiting low CD24 expression appears intriguing. Although the signal networks lying between CD24 and SEC14L2, such as those related to Siglec-10 or metabolism,^16^^,^^29^ is unspecified in OSCC, SEC14L2 neoantigen could be targetable.^32^ The impacts of B cell activity on the immune responses of HNSCC/OSCC have raised extraordinary awareness recently.^39^ Since SEC14L2 is a prognostic predictor of OSCC,^31^ further elucidating the mutation profile of SEC14L2 and the metabolic disruption would precisely define the associated B status and the targeting strategy. miR-146a is one of the dogmas involved in tumorigenesis, stemness, and immune modulation of OSCC.^4^^,^^6^^,^^7^^,^^9^^,^^27^ The reciprocal regulation between miR-146a and CD24 deserves further elucidation.^27^ Testing miR-146a inhibitors in murine preclinical models to insight the potential effects on tumor immunity would enable clinical validity.

This study demonstrates that CD24 facilitates OSCC progression by upregulating SEC14L2 in cells, preclinical models, and dataset analysis. The correlation between CD24 expression and increased Myeloid DC infiltration being found in human OSCC tumors is in concert with the findings in the MOC-L1 cell line. The ubiquitous decrease in the B cell population associated with higher SEC14L2 expression found in HNSCC tumors, which multiple algorithms have retrieved, warrants future mechanistic approaches. The oncogenic progression mediated by this newly identified CD24-SEC14L2 axis would be an intriguing target for OSCC intervention.

## Declaration of competing interest

The authors have no conflicts of interest relevant to this article.

## References

[1] Nakano K. (2021). Progress of molecular targeted therapy for head and neck cancer in clinical aspects. Mol Biomed.

[2] Hübbers C.U., Akgül B. (2015). HPV and cancer of the oral cavity. Virulence.

[3] Hashmi A.A., Mudassir G., Rashid K. (2024). Risk factors of oral squamous cell carcinoma with special emphasis on areca nut usage and its association with clinicopathological parameters and recurrence. Int J Surg Oncol.

[4] Hung P.S., Liu C.J., Chou C.S. (2013). MiR-146a enhances the oncogenicity of oral carcinoma by concomitant targeting of the IRAK11, TRAF6, and Numb genes. PLoS One.

[5] Liu C.J., Tsai M.M., Hung P.S. (2010). MiR-31 ablates expression of the hif regulatory factor FIH to activate the hif pathway in head and neck carcinoma. Cancer Res.

[6] Wang L., Chen Y., Yan Y. (2020). MiR-146a overexpression in oral squamous cell carcinoma potentiates cancer cell migration and invasion possibly via targeting htt. Front Oncol.

[7] Liu Y.T., Yu C.C., Lu M.Y. (2023). MiR-146a participates in the regulation of cancer stemness of oral carcinoma cells. J Dent Sci.

[8] Liu Y.C., Liu S.Y., Lin Y.C., Liu C.J., Chang K.W., Lin S.C. (2024). The disruption of NEAT1-miR-125b-5p-SLC1A5 cascade defines the oncogenicity and differential immune profile in head and neck squamous cell carcinoma. Cell Death Discov.

[9] Taganov K.D., Boldin M.P., Chang K.J. (2006). NF-kappaB-dependent induction of microRNA miR146, an inhibitor targeted to signaling proteins of innate immune responses. Proc Natl Acad Sci U S A.

[10] Chang S.R., Chou C.H., Liu C.J. (2023). The concordant disruption of B7/CD28 immune regulators predicts the prognosis of oral carcinomas. Int J Mol Sci.

[11] Chang S.R., Chou C.H., Tu H.F., Liu C.J., Chang K.W., Lin S.C. (2024). The expression of immune co-stimulators as a prognostic predictor of head and neck squamous cell carcinomas and oral squamous cell carcinomas. J Dent Sci.

[12] Fang X., Zheng P., Tang J. (2010). CD24: from A to Z. Cell Mol Immunol.

[13] Sagiv E., Starr A., Rozovski U. (2008). Targeting CD24 for treatment of colorectal and pancreatic cancer by monoclonal antibodies or small interfering RNA. Cancer Res.

[14] Wang H., Shi P., Shi X. (2023). Surprising magic of CD24 beyond cancer. Front Immunol.

[15] Zhao K., Wu C., Li X. (2024). From mechanism to therapy: the journey of CD24 in cancer. Front Immunol.

[16] Barkal A.A., Brewer R.E., Markovic M. (2019). CD24 signalling through macrophage Siglec-10 is a target for cancer immunotherapy. Nature.

[17] Cui M., Chang Y., Fang Q.G. (2019). Non-coding RNA PVT1 promotes cancer stem cell-like traits in nasopharyngeal cancer via inhibiting miR-1207. Pathol Oncol Res.

[18] Sagiv E., Kazanov D., Arber N. (2006). CD24 plays an important role in the carcinogenesis process of the pancreas. Biomed Pharmacother.

[19] Baumann P., Thiele W., Cremers N. (2012). CD24 interacts with and promotes the activity of c-src within lipid rafts in breast cancer cells, thereby increasing integrin-dependent adhesion. Cell Mol Life Sci.

[20] Salnikov A.V., Bretz N.P., Perne C. (2013). Antibody targeting of CD24 efficiently retards growth and influences cytokine milieu in experimental carcinomas. Br J Cancer.

[21] Bradley C.A. (2019). CD24 - a novel 'don't eat me' signal. Nat Rev Cancer.

[22] Muralidoss H., Muthusekhar M.R. (2023). Expression of CD24 as cancer stem cell marker in the diagnosis of oral squamous cell carcinoma - a prospective study. Ann Maxillofac Surg.

[23] Poothakulath Krishnan R., Pandiar D., Ramani P. (2023). Utility of CD44/CD24 in the outcome and prognosis of oral squamous cell carcinoma: a systematic review. Cureus.

[24] Hemavathy O.R., Marimuthu Ramaswamy M., Mohana Priya C.D. (2025). Role of liquid biopsy in oral premalignant and malignant lesions: correlation with CD24 and CD44 expression in early diagnosis of oral cancer. J Maxillofac Oral Surg.

[25] Zou K.L., Lan Z., Cui H. (2024). CD24 blockade promotes anti-tumor immunity in oral squamous cell carcinoma. Oral Dis.

[26] Fugle C.W., Zhang Y., Hong F. (2016). CD24 blunts oral squamous cancer development and dampens the functional expansion of myeloid-derived suppressor cells. OncoImmunology.

[27] Ghuwalewala S., Ghatak D., Das S. (2021). MiRNA-146a/akt/beta-catenin activation regulates cancer stem cell phenotype in oral squamous cell carcinoma by targeting CD24. Front Oncol.

[28] Li Z., Lou Y., Tian G. (2019). Discovering master regulators in hepatocellular carcinoma: one novel MR, SEC14L2 inhibits cancer cells. Aging (Albany NY).

[29] Liu S., Huang D., Huang J. (2021). Genome-wide expression analysis identifies the association between SEC14L2 and castration-resistant prostate cancer survival. J Cancer.

[30] Song S., Zhang M., Xie P. (2022). Comprehensive analysis of cuproptosis-related genes and tumor microenvironment infiltration characterization in breast cancer. Front Immunol.

[31] David J.J., Kannan B., Pandi C. (2024). Increased SEC14L2 expression is associated with clinicopathological features and worse prognosis in oral squamous cell carcinoma. Odontology.

[32] Sueangoen N., Grove H., Chuangchot N. (2024). Stimulating T cell responses against patient-derived breast cancer cells with neoantigen peptide-loaded peripheral blood mononuclear cells. Cancer Immunol Immunother.

[33] Gossen M., Bujard H. (1992). Tight control of gene expression in mammalian cells by tetracycline-responsive promoters. Proc Natl Acad Sci U S A.

[34] Chang K.W., Lin C.E., Tu H.F. (2020). Establishment of a p53 null murine oral carcinoma cell line and the identification of genetic alterations associated with this carcinoma. Int J Mol Sci.

[35] Chen Y.F., Chang K.W., Yang I.T. (2019). Establishment of syngeneic murine model for oral cancer therapy. Oral Oncol.

[36] Chen Y.F., Liu C.J., Lin L.H. (2019). Establishing of mouse oral carcinoma cell lines derived from transgenic mice and their use as syngeneic tumorigenesis models. BMC Cancer.

[37] Botticelli A., Cirillo A., Strigari L. (2021). Anti-PD-1 and anti-PD-L1 in head and neck cancer: a network meta-analysis. Front Immunol.

[38] Kang J.H., Zappasodi R. (2023). Modulating Treg stability to improve cancer immunotherapy. Trends Cancer.

[39] Li K., Zhang C., Zhou R. (2024). Single cell analysis unveils B cell-dominated immune subtypes in HNSCC for enhanced prognostic and therapeutic stratification. Int J Oral Sci.

