# Supplementary material for: The roles of CD24-Sec14 like lipid binding 2 (SEC14L2) axis in the neoplastic progression of oral squamous cell carcinomas
Source: J Dent Sci. 2025 Aug 22;21(1):232–43. doi: 10.1016/j.jds.2025.08.014 (PMC12825469; doi:10.1016/j.jds.2025.08.014)
Supplement: Multimedia component 1 [file mmc1.docx]

**Supplements**

**Supplementary Tables**

**Table S1.** Reagents

| **Reagent** | **Supplier** | **Cat No.** |
| --- | --- | --- |
| siRNA control | Santa Cruz Biotech | sc-37007 |
| CD24 siRNA | Santa Cruz Biotech | sc-29979 |
| SEC14L2 siRNA | Santa Cruz Biotech | sc-271905 |
| Anti-CD24 Ab (WB) | Thermo Scientific | MA5-11828 |
| Anti-mouse secondary Ab (WB) | Merck Millipore | AP124P |

WB, western blot analysis

**Table S2.** Primers or probes used in this study

1. TaqMan qPCR analysis

| Probes | Supplier | Cat No. |
| --- | --- | --- |
| CD24 | Thermo Scientific | Mm00782538_sH |
| SEC14L2 | Thermo Scientific | Mm00446849_m1 |
| GAPDH | Thermo Scientific | Mm99999915_g1 |

2. CD24 coding sequence

265 bp amplicon (219 bp coding sequence + nucleotides in flanking untranslated regions + enzyme sites + affinity sequence) achieved from the amplification using the following primers:

Forward primer (BamHI site): GCCGGATCCGGACATGGGCAGAGCGATG

Reverse primer (EcoRI site): GCGGAATTCGAGACGTTTCCTGGCCTGAGTC

CGGACATGGGCAGAGCGATGGTGGCCAGGCTAGGGCTGGGGTTGCTGCTTCTGGCACTGCTCCTACCCACGCAGATTTACTGCAACCAAACATCTGTTGCACCGTTTCCCGGTAACCAGAATATTTCTGCTTCCCCAAATCCAAGTAACGCTACCACCAGAGGGGGTGGCAGCTCCCTGCAGTCCACAGCTGGTCTCCTGGCTCTCTCTCTCTCTCTTCTACATCTCTACTGTTAGAGACTCAGGCCAGGAAACGTCTC

Yellow, the tagged primer end; blue and green box, the restriction enzyme cleavage sequence; grey box, CD24 (CD24A) coding sequence; underline, the primer sequence or site

**Table S3.** Antibodies for flow cytometry analysis

| Antibody | Supplier | Cat No. |
| --- | --- | --- |
| APC anti-mouse CD24 | BioLegend | 138505 |
| Anti-mouse SEC14L2 | Novus Biologicals | NBP2-01346 |
| Rhodamine (TRITC) AffiniPure™ Goat Anti-Mouse IgG (H+L) | Jackson ImmunoResearch | 115-025-003 |
| PE anti-mouse CD3e | BD Pharmingen | 553064 |
| APC anti-mouse CD4 | BD Pharmingen | 553051 |
| PE/Cyanine7 anti-mouse CD8a | BioLegend | 100722 |
| Alexa Fluor® 700 anti-mouse Foxp3 | BD Pharmingen | 560401 |
| Brilliant Violet 421™ anti-mouse CD45 | BioLegend | 103134 |
| APC anti-mouse/human CD11b | BioLegend | 101212 |
| Alexa Fluor® 700 anti-mouse F4/80 | BD Pharmingen | 565853 |
| PE/Dazzle™ 594 anti-mouse CD206 (MMR) | BioLegend | 141732 |
| Alexa Fluor® 700 anti-mouse Ly-6C | BioLegend | 128024 |
| PE/Dazzle™ 594 anti-mouse Ly-6G | BioLegend | 127647 |

**Table S4.** The strategies for sorting of immune cell population

**
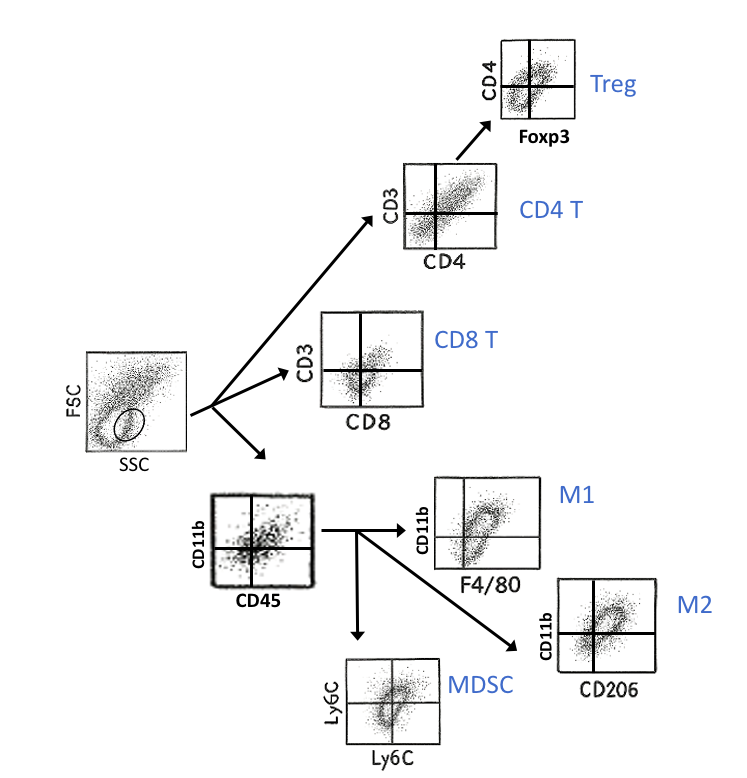
**

**Table S5.** Clinicopathological parameters of our OSCC tumor cohort

| \| Parameters \| *n* = 99 \| \| --- \| --- \| \| Age, years, mean ± SE \| 58.2 ± 9.7 \| \| Sex \|  \| \| Male \| 94 (94.9%) \| \| Female \| 5 (5.0%) \| \| Oral habits \|  \| \| Areca nut chewing \| 63 (77.8%) \| \| Smoking \| 72 (88.9%) \| \| Drinking \| 42 (53.2%) \| \| Site \|  \| \| Buccal mucosa \| 27 (27.3%) \| \| Gingiva \| 31 (31.3%) \| \| Tongue \| 25 (25.3%) \| \| Palate, oropharynx and other sites \| 16 (16.2%) \| \| Differentiation \|  \| \| Well \| 62 (62.6%) \| \| Moderate-poor \| 37 (37.4%) \| \| T classification \|  \| \| T1-3 \| 24 (24.5%) \| \| T4 \| 74 (75.5%) \| \| N classification \|  \| \| N0 \| 54 (55.1%) \| \| N+ \| 44 (44.9%) \| \| TNM classification \|  \| \| I-III \| 21 (21.2%) \| \| IV \| 78 (78.8%) \| \| Follow-up \|  \| \| Disease-free survival \| 43 (43.4%) \| \| Death or recurrence \| 56 (56.6%) \| |  |
| --- | --- | --- | --- | --- | --- | --- | --- | --- | --- | --- | --- | --- | --- | --- | --- | --- | --- | --- | --- | --- | --- | --- | --- | --- | --- | --- | --- | --- | --- | --- | --- | --- | --- | --- | --- | --- | --- | --- | --- | --- | --- | --- | --- | --- | --- | --- | --- | --- | --- | --- | --- | --- | --- | --- | --- | --- | --- | --- | --- |
|  |  |
|  |  |
|  |  |
|  |  |
|  |  |
|  |  |
|  |  |
|  |  |

**Table S6.** Disrupted transcripts in murine OSCC tumors following CD24 expression

**
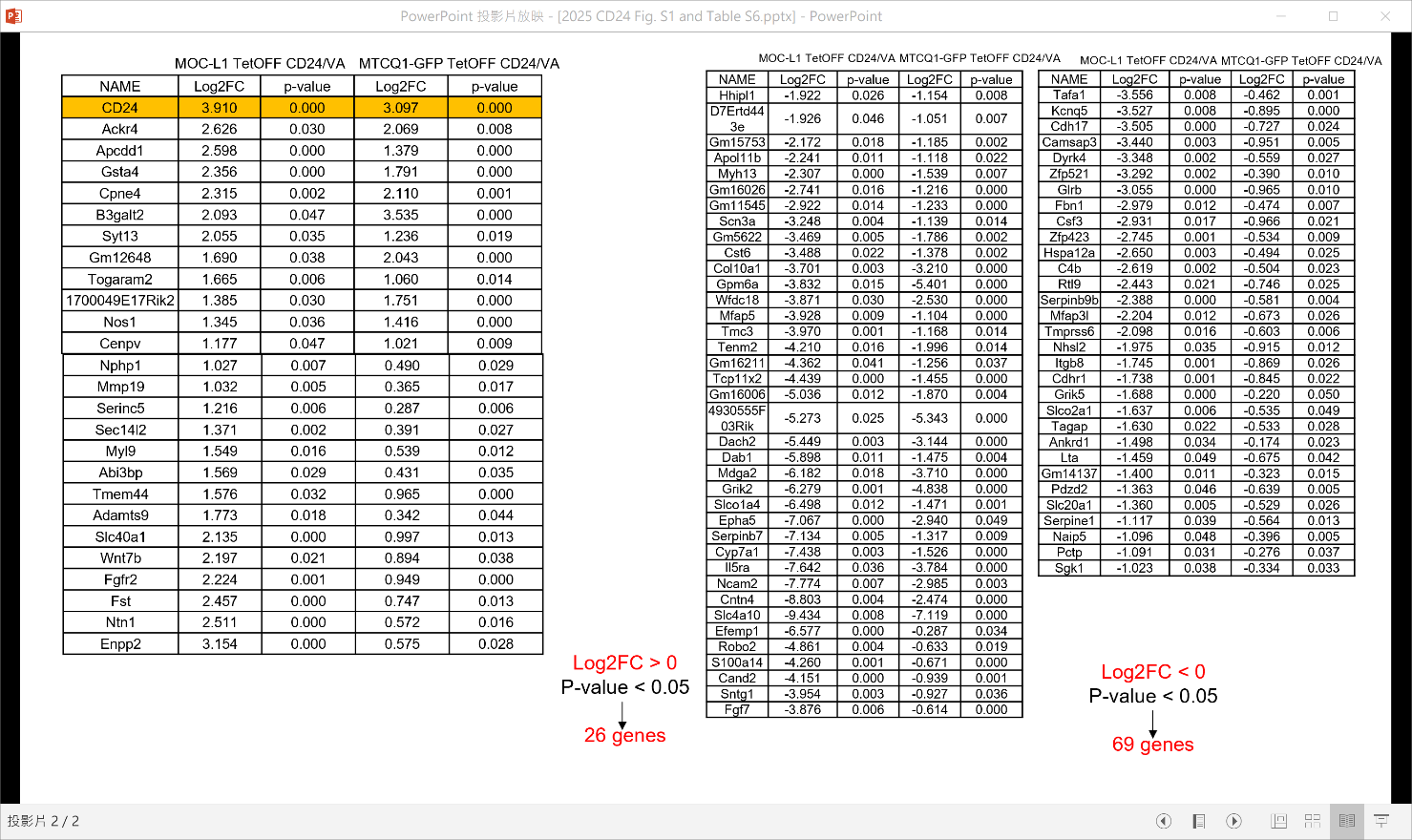
**
